# Single Ascending-Dose Study To Evaluate the Safety, Tolerability, and Pharmacokinetics of Sutezolid in Healthy Adult Subjects

**DOI:** 10.1128/aac.02108-21

**Published:** 2022-03-14

**Authors:** Paul Bruinenberg, Jerry Nedelman, Tian J. Yang, Fran Pappas, Dan Everitt

**Affiliations:** a Global Alliance for TB Drug Development, New York, New York, USA

**Keywords:** sutezolid, tuberculosis, pharmacokinetics, phase 1, safety, antimicrobial safety

## Abstract

The primary objective of the study was to evaluate the safety and tolerability of single oral doses of sutezolid tablets administered under fasting conditions in healthy adult subjects. The secondary objective was to determine the pharmacokinetics (PK) of sutezolid and two metabolites, PNU-101603 and PNU-101244. Overall, sutezolid was well tolerated when administered as a 300-mg, 600-mg, 1,200-mg, or 1,800-mg dose in healthy adult subjects under fasting conditions. Maximum concentration (*C*_max_) of sutezolid, PNU-101603, and PNU-101244 increased in a less-than-proportional manner with an increase in sutezolid dose between 300 mg and 1,800 mg. Total exposure (AUC_last_ [area under the concentration-time curve from time zero to the time of the last quantifiable concentration] and AUC_inf_ [area under the plasma concentration time curve from time zero extrapolated to infinity]) of sutezolid, PNU-101603, and PNU-101244 increased proportionally with an increase in sutezolid dose.

## INTRODUCTION

Tuberculosis (TB) is one of the top 10 causes of death worldwide and the leading cause of death from a single infectious agent in 2019 (1). The World Health Organization (WHO) estimated that 10 million people became ill with TB in 2019, and 1.4 million died (1). An estimated 465,000 incident cases were attributed to rifampicin-resistant TB or multidrug-resistant TB (MDR-TB), which is resistance to both rifampicin and isoniazid (2).

Two main challenges in the treatment of TB disease are the duration and complexity of drug regimens, both of which affect the following: adherence; toxicity, especially of second-line drugs used to treat drug-resistant TB (DR-TB); and the limited availability of pediatric drug formulations for second-line treatment. Current treatment regimens for TB disease require combinations of multiple drugs, ranging from a duration of 6 to 20 months for MDR-TB (3).

While host genetic factors might contribute to the development of MDR-TB, incomplete and inadequate treatment is the most important factor leading to its development (4). Poor adherence to treatment contributes to prolonged infectiousness, drug resistance, relapse, and death (5). Safe, affordable, and easily administered drugs that are effective against DR-TB are urgently needed.

In April 2000, the FDA approved linezolid (Zyvox) to treat serious Gram-positive bacterial infections for up 28 days (6). In recent years, linezolid has been increasingly used to treat difficult cases of MDR-TB and extensively drug-resistant TB (XDR-TB) with apparent clinical benefit (7). However, reports of uncontrolled studies indicate that linezolid’s dose must be reduced often, and/or the duration of its use must be limited due to potentially serious neurologic, ophthalmologic, and hematologic toxicities (8, 9). These toxicities, which typically occur only after months of treatment, are thought to be due to inhibition of mitochondrial protein synthesis (10). Hence, oxazolidinones that may be administered over long periods with reduced toxicity are needed for the treatment of DR-TB.

Sutezolid (PNU-100480), a sulfur-containing oxazolidinone analog of linezolid, has been evaluated as a promising TB drug candidate in preclinical efficacy studies *in vitro* and *in vivo*. Like linezolid, sutezolid inhibits the growth of TB by blocking microbial RNA translation and, thereby, protein synthesis, and it appears to be effective against MDR-TB and XDR-TB (11). Because bacterial protein synthesis is not targeted by any of the drugs in the first-line standard of care for treatment of tuberculosis (isoniazid, rifampicin, pyrazinamide, ethambutol), oxazolidinones have no known preexisting resistance or cross-resistance with other TB drug classes (12).

Sutezolid is metabolized to an active sulfoxide metabolite (PNU-101603) and another active but much less prevalent sulfone metabolite (PNU-101244) by flavin-containing monooxygenases (13). In mice dosed with 100 mg/kg once a day (QD), the sulfoxide metabolite achieved five and 10 times the exposure of the parent on the 1st and 24th days of dosing, respectively (14). In cynomolgus macaques, sulfoxide exposure was approximately four times that of parent (15). A one-compartment model for parent with an additional compartment for the sulfoxide characterized the cynomolgus data, although different parameter estimates were reported for 20 mg/kg and 40 mg/kg QD versus 40 mg/kg twice-a-day (BID) dosing (15).

Plasma protein binding of sutezolid, the sulfoxide, and the sulfone is 48%, 4%, and 6%, respectively, at 1 μg/mL (16).

Prior clinical studies have been conducted with sutezolid manufactured by Pfizer. Between 2009 and 2011, Pfizer tested sutezolid in healthy human subjects in a single-ascending-dose (SAD) study (16) and a multiple-ascending-dose (MAD) study (17) and in sputum smear-positive tuberculosis patients in an early bactericidal activity (EBA) study (12). The SAD study made use of a Pfizer suspension formulation, whereas the MAD study was conducted with the company’s suspension and tablet formulation. The EBA study was conducted with the Pfizer tablet formulation. No significant adverse events (AEs) were observed in the SAD study (up to 1,500 mg), MAD study (up to 1,200 mg once daily for 14 days and 600 mg twice daily for 28 days), and EBA study (600 mg twice daily and 1,200 mg once daily for 14 days). There was no effect on the QT interval. However, in the EBA study, seven sutezolid-treated patients (14%) had transient, asymptomatic alanine transaminase (ALT) elevations to 173 ± 34 U/L on day 15, none met Hy’s criteria for serious liver injury, and values for all patients had returned to normal on day 42.

Studies of novel regimens in mice have shown that sutezolid contributes at least as much as linezolid to bactericidal activity (18). Therefore, it has the potential to be a replacement for linezolid.

TB Alliance has developed a new synthetic drug-substance process and tablet formulation of sutezolid in 100-mg and 600-mg strengths. These tablets have been release-tested via International Council for Harmonization (ICH) guidelines. A randomized, double-blind, placebo-controlled, SAD study has been conducted to evaluate the safety, tolerability, and pharmacokinetics (PK) of single oral doses of these new tablets in healthy adult subjects under fasting conditions. The objective here is to describe the results of that study and thereby characterize the properties of the new sutezolid tablets.

## RESULTS

### Safety.

Overall, sutezolid was well tolerated when administered as a single 300-mg, 600-mg, 1,200-mg, or 1,800-mg dose in healthy adult subjects under fasting conditions. There were no serious AEs or AEs that led to subject discontinuation; all subjects completed the study. Twenty-one of 32 subjects (65.6%) reported a total of 30 treatment-emergent AEs (TEAEs), all mild. Eight (25.0%) subjects reported nine TEAEs that the investigator considered possibly or probably treatment related. There were no significant electrocardiogram (ECG) changes, and no AEs related to clinically significant ECGs or physical examinations. The number of subjects with TEAEs and the number of subjects with TEAEs assessed by the investigator as related to study treatment are presented by treatment group in Table 1. The treatment-related TEAEs are further identified in Table 2.

**TABLE 1:** Subjects with TEAEs and TEAEs related to study treatment following single dose of placebo or sutezolid^*a*^

MedDRA primary system organ, class/preferred term	Placebo (*n* = 8)	Sutezolid, 300 mg (*n* = 6)	Sutezolid, 600 mg (*n* = 6)	Sutezolid, 1,200 mg (*n* = 6)	Sutezolid, 1,800 mg (*n* = 6)	Overall (*n* = 32)
Subjects with any TEAEs, *n* (%)	5 (62.5)	4 (66.7)	5 (83.3)	3 (50.0)	4 (66.7)	21 (65.6)
Subjects with any treatment-related^*b*^ TEAEs, *n* (%)	2 (25.0)	1 (16.7)	0	2 (33.3)	3 (50.0)	8 (25.0)

a Each cohort consisted of six subjects who took sutezolid and two subjects who took placebo. There were no serious AEs and no AEs that led to subject discontinuation. All events were of mild intensity. *n*, number of subjects; AE, adverse event; TEAE, treatment-emergent AE.

b Possibly or probably related.

**TABLE 2:** Treatment-emergent adverse events assessed as related to study treatment by the blinded site investigator following a single dose of placebo or sutezolid^*a*^

Preferred term	Placebo (*n* = 8)	Sutezolid, 300 mg (*n* = 6)	Sutezolid, 600 mg (*n* = 6)	Sutezolid, 1,200 mg (*n* = 6)	Sutezolid, 1,800 mg (*n* = 6)
Abdominal distension	0	0	0	0	1
Abdominal pain	0	0	0	1	0
Myalgia	0	0	0	0	1
Postural orthostatic tachycardia syndrome	2	1	0	1	1
Transaminases increased	0	0	0	1	0

a Each cohort consisted of six subjects who took active drug (sutezolid) and two subjects who took placebo. *n* = number of subjects administered placebo or sutezolid in each dosing group.

One subject who received 1,200 mg sutezolid experienced elevated transaminases. Aspartate transaminase (AST) values on days −1, 2, and 3 were 28, 25, and 35 U/L, respectively, with an upper limit of normal (ULN) of 32 U/L. ALT values were 47, 42, and 51 U/L, respectively, with a ULN of 44 U/L. When the subject returned for an unscheduled follow-up visit on day 6, values were within the normal range, 27 and 43 U/L for AST and ALT, respectively.

### PK results.

The study enrolled 32 healthy adult subjects, 24 who received study drug and 8 who received placebo. Data from all 24 subjects who received active treatment were included in the PK analyses.

**FIG 1:**
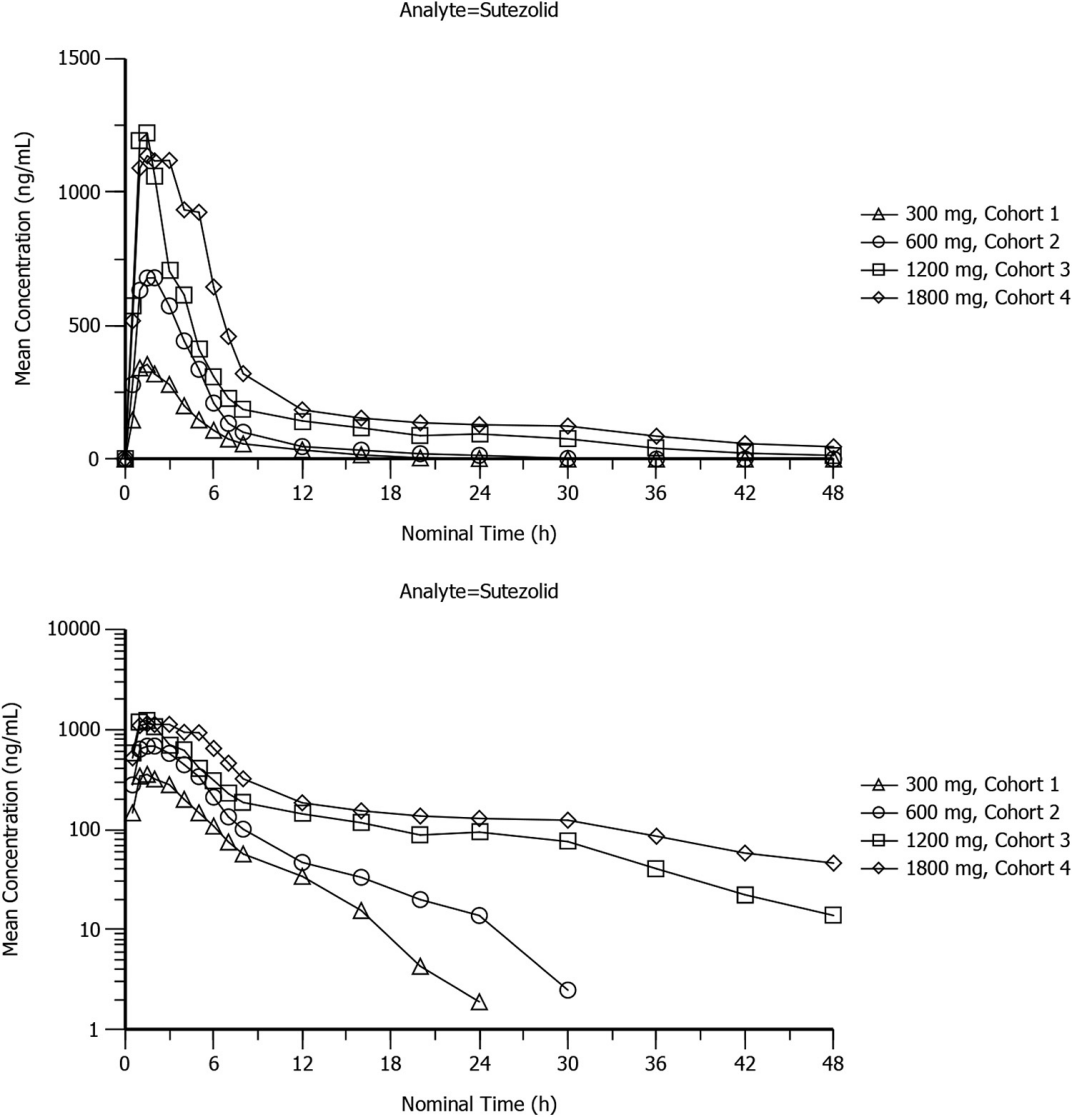
Mean plasma concentration-time profiles of sutezolid after single doses of 300-mg (cohort 1), 600-mg (cohort 2), 1,200-mg (cohort 3), and 1,800-mg (cohort 4) sutezolid tablets on linear and semilogarithmic scales (top and bottom panels, respectively).

**FIG 2:**
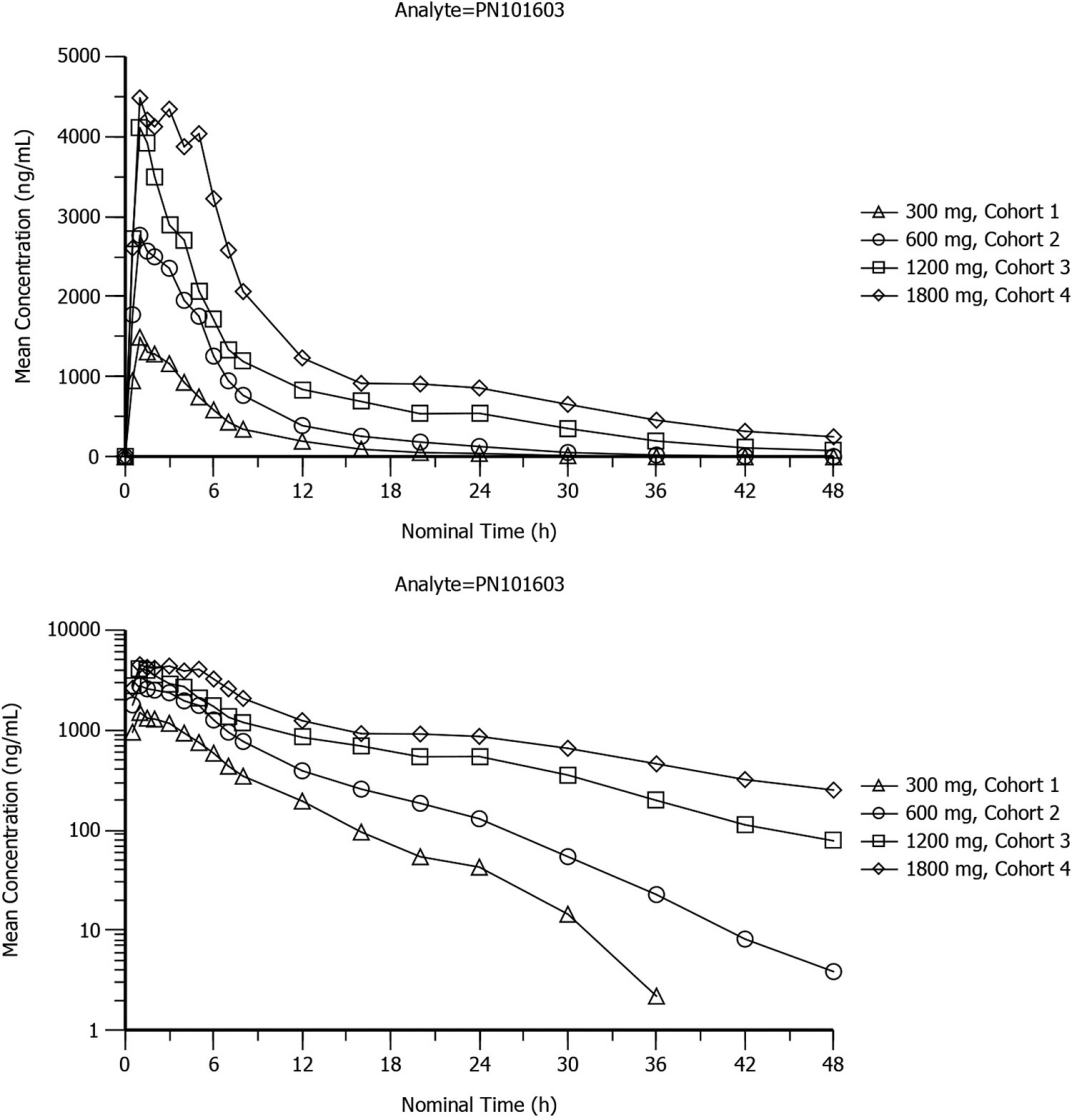
Mean plasma concentration-time profiles of PNU-101603 after single doses of 300-mg (cohort 1), 600-mg (cohort 2), 1,200-mg (cohort 3), and 1,800-mg (cohort 4) sutezolid tablets on linear and semilogarithmic scales (top and bottom panels, respectively).

Mean plasma concentration-time profiles of sutezolid, PNU-101603, and PNU-10244 after single doses of 300 mg, 600 mg, 1,200 mg, and 1,800 mg sutezolid tablets are plotted on linear and semilogarithmic scales in Fig. 1, 2, and 3. Individual curves for sutezolid and its metabolites are provided in Fig. S1 and S2 in the supplemental material. Mean concentration curves of sutezolid and PNU-101603 exhibited multiphasic distribution/elimination, more prominently at the higher two doses, with a rapid decline after the peak followed by a somewhat prolonged plateau and then a return to a steeper slope. Such a pattern is also hinted at for PNU-10244, where many concentrations were below the limit of quantification (BLQ).

**FIG 3:**
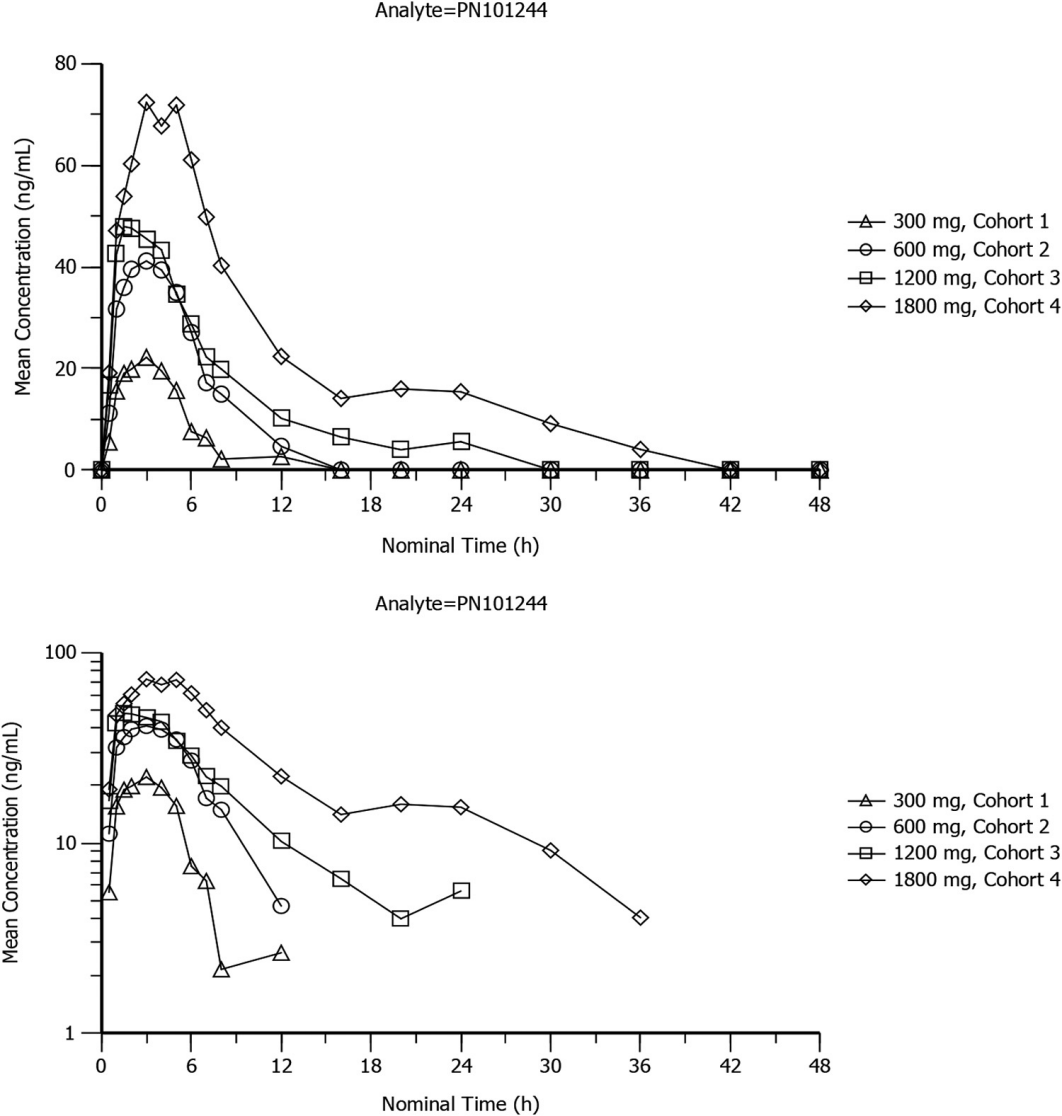
Mean plasma concentration-time profiles of PNU-101244 after single doses of 300-mg (cohort 1), 600-mg (cohort 2), 1,200-mg (cohort 3), and 1,800-mg (cohort 4) sutezolid tablets on linear and semilogarithmic scales (top and bottom panels, respectively).

The PK parameters of sutezolid and PNU-101603 determined in this study are summarized below in Tables 3 and 4. See Table S1 in the supplemental material for a summary of plasma PK parameters of PNU-101224.

**TABLE 3:** Plasma pharmacokinetic parameters of sutezolid

Parameter	Cohort 1 (300 mg)				Cohort 2 (600 mg)				Cohort 3 (1,200 mg)				Cohort 4 (1,800 mg)			
	*n*	Mean	SD	CV%	*n*	Mean	SD	CV%	*n*	Mean	SD	CV%	*n*	Mean	SD	CV%
*T*_max_ (h)	6	2.18	0.94	43.15	6	2.25	1.54	68.49	6	1.75	1.13	64.52	6	2.50	1.52	60.66
*C*_max_ (ng/mL)	6	408	109	26.81	6	782	211	27.06	6	1,440	571	39.62	6	1,550	633	40.85
AUC_last_ (h · ng/mL)	6	1,810	490	27.11	6	3,690	694	18.80	6	7,720	3,060	39.63	6	11,400	5,350	47.08
AUC_inf_ (h · ng/mL)	6	1,880	482	25.56	6	3,810	686	18.01	6	8,020	3,160	39.45	5	14,100	5,880	41.66
AUC_extrap_ (%)	6	4.43	1.90	42.90	6	3.14	1.12	35.52	6	3.80	1.64	43.17	5	7.23	6.31	87.33
λ*_z_* (h^−1^)	6	0.175	0.0308	17.67	6	0.134	0.0585	43.59	6	0.0851	0.0194	22.83	5	0.0774	0.0442	57.13
*t*_1/2_ (h)	6	4.08	0.773	18.93	6	5.74	1.61	28.00	6	8.48	1.79	21.07	5	11.7	6.80	58.05
*T*_last_ (h)	6	17.3	4.13	23.83	6	23.7	4.46	18.83	6	40.0	9.03	22.58	6	42.0	9.30	22.13
*C*_last_ (ng/mL)	6	13.2	1.31	9.92	6	14.0	2.45	17.53	6	23.8	11.6	48.84	6	51.2	42.0	81.99
CL/F (L/h)	6	167	38.2	22.83	6	161	27.1	16.78	6	167	54.8	32.89	5	145	55.4	38.25
*V_z_*/F (L)	6	990	335	33.82	6	1,360	436	32.09	6	2,060	898	43.60	5	2,040	382	18.71

**TABLE 4:** Plasma pharmacokinetic parameters of PNU-101603

Parameter	Cohort 1 (300 mg)				Cohort 2 (600 mg)				Cohort 3 (1,200 mg)				Cohort 4 (1,800 mg)			
	*n*	Mean	SD	CV%	*n*	Mean	SD	CV%	*n*	Mean	SD	CV%	*n*	Mean	SD	CV%
*T*_max_ (h)	6	1.68	1.05	62.60	6	2.33	1.72	73.82	6	1.50	1.26	84.33	6	2.50	1.79	71.55
*C*_max_ (ng/mL)	6	1,620	513	31.64	6	3,070	782	25.50	6	4,770	858	17.99	6	5,850	1,730	29.51
AUC_last_ (h · ng/mL)	6	9,210	2,550	27.73	6	20,100	4,730	23.55	6	36,900	2,070	5.60	6	57,800	12,800	22.16
AUC_inf_ (h · ng/mL)	6	9,320	2,580	27.67	6	20,200	4,730	23.42	6	38,200	3,340	8.75	6	64,100	19,300	30.17
AUC_extrap_ (%)	6	1.21	0.327	27.03	6	0.595	0.187	31.38	6	3.04	3.56	117.05	6	7.90	7.99	101.18
λ*_z_* (h^−1^)	6	0.159	0.0360	22.65	6	0.149	0.0484	32.44	6	0.117	0.0579	49.31	6	0.0889	0.0623	70.05
*t*_1/2_ (h)	6	4.54	0.919	20.26	6	5.07	1.62	31.90	6	7.61	4.42	58.04	6	11.9	8.09	68.00
*T*_last_ (h)	6	29.0	4.50	15.52	6	39.0	9.10	23.33	6	47.0	2.45	5.21	6	47.0	2.45	5.21
*C*_last_ (ng/mL)	6	16.9	3.58	21.19	6	16.7	5.67	33.97	6	80.3	75.3	93.80	6	253	219	86.76

### Sutezolid.

As dose increased from 300 mg to 1,800 mg, the mean sutezolid *C*_max_ (maximum concentration of drug in plasma) values increased from 408 ng/mL to 1,550 ng/mL, and mean area under the concentration-time curve from time zero extrapolated to infinity (AUC_inf_) increased from 1,880 h · ng/mL to 14,100 h · ng/mL. Mean values of *T*_max_ (time to maximum concentration of drug in plasma) ranged between 1.75 h and 2.50 h postdose.

Mean sutezolid clearance (CL/F) values were similar across cohorts, ranging from 145 L/h to 167 L/h, with no dose dependence. But mean half-life (*t*_1/2_) increased with dose from 4.08 h to 11.7 h, and mean volume of distribution (*V_z_*/F) increased from 990 L at 300 mg to around 2,000 L at both 1,200 and 1,800 mg. Differences observed in *t*_1/2_ and *V_z_*/F values reflect the greater elaboration of the central plateau portion of the elimination phase at higher doses.

Variability (percent coefficient of variation [CV%]) in maximum and total sutezolid exposure was lower after 300-mg and 600-mg doses (18% to 27%) compared to after 1,200-mg and 1,800-mg doses (39% to 47%). Similarly, variability of *t*_1/2_ increased with dose. These patterns reflect the increase of variability in the individual concentration profiles evident in Fig. S1 and S2 in the supplemental material.

Results of the power model for assessing dose proportionality for sutezolid are summarized in Table 5. The slope value for *C*_max_ was 0.74, and the 90% confidence interval did not include 1 (0.56, 0.93). The mean *C*_max_ values seem consistent with dose proportionality through 1,200 mg, approximately doubling as the doses doubled, but with a lower mean *C*_max_ at 1,800 mg than continued dose proportionality would predict. Slopes for AUC_last_ (area under the concentration-time curve from time zero to the time of the last quantifiable concentration) and AUC_inf_ were 0.99 and 1.08, respectively, and 90% confidence intervals included 1 for both parameters: AUC_last_ (0.81, 1.17) and AUC_inf_ (0.92, 1.24).

**TABLE 5:** Assessment of dose proportionality of sutezolid following single-dose administration of sutezolid^*c*^

Dependent variable	Model variable	Estimate (β_1_)	Lower CI^*a*^	Upper CI^*a*^	Rho_1_^*b*^
ln(*C*_max_)	ln(dose)	0.7449	0.5613	0.9285	1.9266
ln(AUC_last_)	ln(dose)	0.9878	0.8098	1.1658	4.5390
ln(AUC_inf_)	ln(dose)	1.0791	0.9242	1.2340	3.4200

a 90% confidence intervals (lower and upper).

b High/low dose ratio in which dose proportionality can be demonstrated definitely, relative to the lowest dose in the analysis data set. Rho_1_ was calculated as Rho_1_ = (ϴ_H_) ^ (1/max(1 − lower, upper − 1)), in which ϴ_H_ = 1.333.

c Power model: ln(PK) = ln(β_0_) + β_1_ × ln(dose) + ε, where PK is the pharmacokinetic parameter tested, ln(β_0_) is the *y*-intercept, β_1_ is the slope, and ε is an error term.

### PNU-101603.

Mean PNU-101603 maximum and total exposure increased with increases in sutezolid dose, *C*_max_ ranging from 1,620 ng/mL to 5,850 ng/mL and AUC_inf_ ranging from 9,320 h · ng/mL to 64,100 h · ng/mL. AUC_inf_ values for PNU-101603 were around five times the values for sutezolid at each dose. Mean values of *T*_max_ ranged between 1.50 h and 2.50 h postdose. CV% was consistently below around 30%. As was observed for sutezolid, and for the same reason, mean PNU-101603 *t*_1/2_ increased with dose, from 4.54 h to 11.9 h (1,800 mg). As with sutezolid, *C*_max_ increased in a less-than-proportional manner, while AUC_last_ and AUC_inf_ increased proportionally with dose (see Table S2 in the supplemental material).

### PNU-101244.

AUC_inf_ values for PNU-101244 were around 2% of the values for sutezolid at each dose. As with the other two analytes, *C*_max_ increased in a less-than-proportional manner, while AUC_last_ and AUC_inf_ increased proportionally with dose (see Table S3 in the supplemental material).

## DISCUSSION

Overall, sutezolid was found to be safe and well tolerated when administered as single doses of the TB Alliance tablet at 300 mg, 600 mg, 1,200 mg, or 1,800 mg in healthy adult subjects under fasting conditions. In particular, the safety and PK results encompassed the 1,800-mg dose, a dose level not previously tested in a clinical study with sutezolid.

Maximum sutezolid, PNU-101603, and PNU-101244 exposure (*C*_max_) increased in a less-than-proportional manner with an increase in sutezolid dose between 300 mg and 1,800 mg. Total sutezolid, PNU-101603, and PNU-101244 exposure (AUC_last_ and AUC_inf_) increased proportionally with an increase in sutezolid dose.

The dose-proportional increases in AUC, the less-than-proportional increases in *C*_max_, and the complex and variable concentration-profile shapes suggest the possibility that, at least under fasting conditions, as sutezolid dose increases, absorption saturates in the upper gastrointestinal (GI) tract but is eventually completed more slowly through the lower GI tract.

In the SAD study (16) performed by Pfizer with a suspension formulation under fasting conditions, *C*_max_ of sutezolid increased approximately proportionally between 300 mg and 1,000 mg but then declined at 1,500 mg, and similarly for the sulfoxide. Results were not reported for AUCs. Pfizer then conducted a MAD study of the suspension formulation under both fed and fasting conditions in four cohorts: 100 mg BID (fasted), 300 mg BID (fasted), 600 mg BID (fed), and 1,200 mg QD (fed). As shown in Table 6 below, AUC_inf_ from the present study was comparable with AUC_tau_ of the Pfizer study in the 300-mg, 600-mg, and 1,200-mg cohorts. And *C*_max_ and AUC in Pfizer’s MAD study exhibited approximately dose-proportional behavior, unlike in the company’s SAD study. Thus, it may be the case that the fed conditions at the higher doses of Pfizer’s MAD study facilitated more complete, early absorption. However, *t*_1/2_ in the present study (4 to 12 h) was longer than that observed in the Pfizer study (3 h). This may be because Pfizer computed *t*_1/2_ using only concentrations through 12 or 24 h postdose for the multiple-dose BID or QD treatment arms, respectively. A more relevant metric may be the effective *t*_1/2_ determined from the accumulation ratio Rac. As shown in Table 6, values of effective *t*_1/2_ from the Pfizer MAD study range from 3 to 8 h, more in the range of values of *t*_1/2_ from the present study.

**TABLE 6:** Selected sutezolid PK parameters of the TB Alliance SAD study compared with PK parameters of Pfizer MAD study

Pfizer MAD^*g*^	100 mg BID (*n* = 8) (suspension fasted)	300 mg BID (*n* = 8) (suspension fasted)	600 mg BID (*n* = 7) (suspension fed)	1,200 mg QD (*n* = 8) (suspension fed)	
AUC_tau_ (ng · h/mL)^*a*^	845.8 (43)	2,133 (23)	4,294 (23)	10,100 (30)	
*C*_max_ (ng/mL)^*a*^	252 (43)	459 (45)	943 (20)	2,020 (50)	
*T*_max_ (h)^*b*^	1.0 (0.5–2.0)	2.0 (0.5–2.0)	3.0 (2.0–3.0)	3.0 (2.0–3.0)	
*t*_1/2_ (h)^*c*^^,^^*d*^	2.72 (5)	2.55 (27)	2.92 (41)	3.38 (10)	
Rac^*a*^^,^^*e*^	1.565 (12)	1.080 (19)	1.194 (14)	1.051 (14)	
Effective *t*_1/2_^*f*^	8.16	3.20	4.58	5.50	

TBA^*h*^ SAD	300 mg single (tablet fasted)	600 mg single (tablet fasted)	1,200 mg single (tablet fasted)	1,800 mg single (tablet fasted)
AUC_0–inf_ (ng · h/mL)^*a*^	1,840 (25)	3,760 (18)	7,580 (37)	13,200 (42)
*C*_max_ (ng/mL)^*a*^	396 (26)	759 (27)	1,350 (41)	1,430 (50)
*T*_max_ (h)^*b*^	2.3 (1.0–3.1)	1.8 (1.0–5.0)	1.5 (1.0–4.0)	2.5 (1.0–5.0)
*t*_1/2_ (h)^*c*^^,^^*d*^	4.08 (19)	5.74 (36)	8.48 (22)	11.7 (64)

a Data represent the geometric mean (percent coefficient of variation).

b Data represent the median (range).

c Data represent the arithmetic mean (percent coefficient of variation).

d *t*_1/2_ = half-life.

e Rac = accumulation ratio, AUC_0–tau, Day 14_/AUC_0–tau, Day 1_, where tau = 12 h for BID and tau = 24 h for QD.

f Effective *t*_1/2_ computed from the geometric mean Rac as −log(2) × tau/log((Rac − 1)/Rac).

g All results for the Pfizer MAD study are from reference 14 except for effective *t*_1/2_.

h TBA, TB Alliance.

Results from this first study of the TB Alliance tablet formulation of sutezolid support further investigation of multiple doses and of safety and efficacy in patients.

## MATERIALS AND METHODS

### Study design.

The protocol and other study documents, including the informed consent document, were reviewed and approved by the institutional review board (IRB), IntegReview IRB, located in Austin, TX. The study was conducted by Worldwide Clinical Trials Early Phase Services, LLC, at a single clinical site in San Antonio, TX.

Four cohorts of healthy subjects (*n* = 8 each) were randomly assigned at a 3:1 ratio to receive sutezolid tablet (*n* = 6) or placebo (*n* =2) under fasting conditions. The number of subjects selected for the study was based on the number considered adequate to provide sufficient safety data; this study was not formally powered. Cohort 1 was separated into two groups: a sentinel group of 3 subjects (2 sutezolid and 1 placebo) were dosed at least 24 h before the remaining 5 subjects (4 sutezolid and 1 placebo). All subjects in cohort 1 received a single dose of 300 mg sutezolid or placebo. Subjects in the other three cohorts received placebo or single ascending doses of sutezolid (600 mg, 1,200 mg, or 1,800 mg). Each subject participated in only 1 dose level. The study enrolled subjects who were healthy males or females of nonchildbearing potential, between the ages of 19 and 50 years (inclusive), with a body mass index of ≥18.5 and ≤32.0 kg/m^2^, and a body weight of at least 50.0 kg.

Dose escalation to the next cohort (dose level) did not take place until the sponsor, in conjunction with the principal investigator, determined that adequate safety, tolerability, and PK data from the previous cohorts demonstrated confidence to proceed.

Blood was collected for PK analysis prior to study drug administration and at serial time points through 48 h after study drug administration. Blood and urine were collected for clinical laboratory evaluations. Female subjects had blood collected for serum pregnancy testing. Postmenopausal females had blood collected to measure follicle-stimulating hormone (FSH) levels. Subjects were housed in the clinic from at least 24 h prior (day −1) until 48 h (day 3) after dosing. Subjects were contacted via a phone call for follow-up questioning about AEs on day 10 (+1 day).

### Criteria for evaluation. (i) Safety.

The investigator evaluated safety using the following assessments: physical and neurological examinations, vital signs, ECGs, cardiac monitoring, clinical laboratory tests (including hematology, serum chemistry, coagulation, and urinalysis), and reported or observed AEs.

### (ii) Pharmacokinetics.

Blood samples for determination of plasma concentrations of sutezolid, PNU-101603, and PNU-101244 were collected at predose (0 h) and at 0.5, 1, 1.5, 2, 3, 4, 5, 6, 7, 8, 12, 16, 20, 24, 30, 36, 42, and 48 h postdose in each treatment period.

Blood was collected into two prechilled 6-mL Vacutainer tubes containing K2-EDTA, gently inverted 8 to 10 times, and immediately placed on wet ice. Samples were maintained on wet ice throughout processing. Within 60 min of blood collection, samples were centrifuged at approximately 1,500 × *g* at approximately 4°C (±10°C) for 10 min. After centrifugation, plasma was harvested and transferred in approximately equal amounts into four appropriately labeled 5-mL amber cryovials and immediately placed on dry ice. The two primary aliquots contained at least 0.5 mL of plasma. Within 90 min of blood sample collection, aliquots were stored upright in a freezer set at approximately −20°C (±10°C) until transferred on dry ice to the Alliance Pharma, Inc., bioanalytical laboratory for analysis.

Plasma samples were analyzed using a validated LC-MS/MS (liquid chromatography-tandem mass spectrometry) method. Accuracy for all three analytes ranged from 96.4% to 105.5% over 10 to 10,000 ng/mL. Of reassayed samples, 95.7% to 100% met acceptance criteria.

Concentration-time data that were below the limit of quantification (BLQ) were treated as zero in the data summarization and descriptive statistics. During the pharmacokinetic analysis, concentrations that were BLQ up to the time of the first quantifiable concentration were treated as zero. Embedded (values between 2 quantifiable concentrations) and/or terminal BLQ concentrations were treated as “missing.” Actual sample times were used for all pharmacokinetic and statistical analysis. The PK analysis set (all subjects with sufficient data to derive PK parameters) was used in the pharmacokinetic and statistical analyses.

The following PK parameters were calculated for sutezolid, PNU-101603, and PNU-101244, using default settings in WinNonlin except where noted: peak concentration in plasma (*C*_max_), time to peak concentration (*T*_max_), elimination rate constant (λ*_z_*), terminal half-life (*t*_1/2_), area under the concentration-time curve from time zero to the time of the last quantifiable concentration (AUC_last_, using the linear trapezoidal rule), area under the plasma concentration time curve from time zero extrapolated to infinity (AUC_inf_ = AUC_last_ + *C*_last_/λ*_z_*), percentage of AUC_inf_ based on extrapolation (AUC_extrap_), apparent total body clearance (CL/F; calculated for sutezolid only), apparent volume of distribution (*V_z_*/F; calculated for sutezolid only), the last quantifiable concentration (*C*_last_), and the time of the last quantifiable concentration (*T*_last_).

### (iii) Dose proportionality.

The PK parameters *C*_max_, AUC_last_, and AUC_inf_ for sutezolid and metabolites were compared across doses to assess dose proportionality. Statistical analyses were done using a power model (19) of the following general form:

$$\text{ln}\left(\text{PK}\right)=\text{ln}\left(\beta_{0}\right)+\beta_{1}\cdot \text{ln}\left(\text{dose}\right)+\varepsilon$$

where PK is the PK parameter tested (e.g., *C*_max_ or AUC), ln(β_0_) is the *y*-intercept, β_1_ is the slope (a value of β_1_ ≈ 1 indicates dose proportionality), and ε is an error term. The estimate of β_1_ with the 90% confidence interval and the dose range for proportionality were reported. Statistical analysis was performed in SAS (Version 9.4, SAS Institute Inc.) using the PROC MIXED procedure.

### Data availability.

TB Alliance is committed to making sutezolid data and drug material available to the research community and has established an independent review committee to facilitate requests for access. Please contact SIRCquery@tballiance.org for additional information.
