# Supplementary material for: Single Ascending-Dose Study To Evaluate the Safety, Tolerability, and Pharmacokinetics of Sutezolid in Healthy Adult Subjects
Source: Antimicrob Agents Chemother. 2022 Mar 14;66(4):e02108-21. doi: 10.1128/aac.02108-21 (PMC9017382; doi:10.1128/aac.02108-21)

Single Ascending Dose Study to Evaluate the Safety, Tolerability, and Pharmacokinetics of Sutezolid in Healthy Adult Subjects

Authors: Paul Bruinenberg,[1*] Jerry Nedelman,[1] Tian J Yang, [1] Fran Pappas, [1] Dan Everitt[1]

Global Alliance for TB Drug Development, New York, New York

*Corresponding author. Mailing address: 40 Wall Street, 24th Floor, New York NY 10005.

Phone: (212) 227-7540. E-mail: Paul.Bruinenberg@tballiance.org

Supplementary Materials

Supplementary Table S1: Plasma Pharmacokinetic Parameters of PNU-101244

Parameter	Cohort 1: (300 mg) n	Mean	SD	CV%	Cohort 2: (600 mg) n	Mean	SD	CV%
T_{max} (h)	6	2.51	0.85	33.65	6	3.17	1.17	36.92
C_{max} (ng/mL)	6	24.5	7.84	31.97	6	46.9	15.8	33.59
AUC_{last} (h*ng/mL)	6	117	52.7	44.95	6	263	142	54.23
AUC_{inf} (h*ng/mL)	3	219	80.8	36.86	6	330	169	51.31
AUC_{Extrap} (%)	3	42.9	23.5	54.83	6	21.3	5.95	27.95
λ_z (h^{-1})	3	0.189	0.109	57.77	6	0.238	0.0741	31.08
$T_{1/2}$ (h)	3	4.91	3.37	68.73	6	3.18	1.11	35.05
T_{last} (h)	6	7.17	2.64	36.83	6	8.67	2.73	31.53
C_{last} (ng/mL)	6	14.6	3.72	25.49	6	14.3	3.49	24.48
**Parameter**	**Cohort 3: (1200 mg) n**	**Mean**	**SD**	**CV%**	**Cohort 4: (1800 mg) n**	**Mean**	**SD**	**CV%**
T_{max} (h)	6	2.09	1.16	55.44	6	3.25	1.33	40.99
C_{max} (ng/mL)	6	55.8	8.40	15.04	6	85.0	33.7	39.66
AUC_{last} (h*ng/mL)	6	402	129	32.18	6	877	262	29.94
AUC_{inf} (h*ng/mL)	5	562	292	51.99	3	1250	354	28.27
AUC_{Extrap} (%)	5	26.3	13.8	52.63	3	17.4	4.70	27.00
λ_z (h^{-1})	5	0.154	0.125	81.02	3	0.0596	0.0315	52.74
$T_{1/2}$ (h)	5	10.6	10.5	98.87	3	13.6	5.57	41.04
T_{last} (h)	6	16.7	8.16	48.99	6	29.3	6.41	21.85
C_{last} (ng/mL)	6	11.9	1.27	10.69	6	11.8	1.56	13.21

Supplementary Table S2: Assessment of Dose Proportionality of PNU-101603 Following Single Dose Administration of Sutezolid

Dependent Variable	Model Variable	Estimate (β1)	Lower CI[a]	Upper CI[a]	Rho1[b]
**ln(C_{max})**	ln(Dose)	0.7217	0.5875	0.8559	2.0087
**ln(AUC_{last})**	ln(Dose)	1.0183	0.9071	1.1294	9.2440
**ln(AUC_{inf})**	ln(Dose)	1.0559	0.9334	1.1783	5.0193

Power Model: $\ln(PK)=\ln(\beta_0)+\beta_1*\ln(Dose)+\varepsilon$, where PK is the pharmacokinetic parameter tested, $\ln(\beta_0)$ is the y-intercept, β_1 is the slope, and ε is an error term.

[a] 90% confidence intervals (lower and upper).

[b] High/low dose ratio in which dose proportionality can be demonstrated definitely, relative to the lowest dose in the analysis dataset. Rho_1 was calculated as: $Rho_1=(\Theta_H)$^(1/max(1-lower, upper-1)), in which $\Theta_H=1.333$.

Supplementary Table S3: Assessment of Dose Proportionality of PNU-101244 Following Single Dose Administration of Sutezolid

Dependent Variable	Model Variable	Estimate (β1)	Lower CI[a]	Upper CI[a]	Rho1[b]
**ln(C_{max})**	ln(Dose)	0.6348	0.4726	0.7970	1.7254
**ln(AUC_{last})**	ln(Dose)	1.0811	0.8575	1.3046	2.5714
**ln(AUC_{inf})**	ln(Dose)	0.9081	0.5601	1.2561	1.9233

Power Model: $\ln(PK)=\ln(\beta_0)+\beta_1*\ln(Dose)+\varepsilon$, where PK is the pharmacokinetic parameter tested, $\ln(\beta_0)$ is the y-intercept, β_1 is the slope, and ε is an error term.

[a] 90% confidence intervals (lower and upper).

[b] High/low dose ratio in which dose proportionality can be demonstrated definitely, relative to the lowest dose in the analysis dataset. Rho_1 was calculated as: $Rho_1=(\Theta_H)$^(1/max(1-lower, upper-1)), in which $\Theta_H=1.333$.

Supplementary Figure S1: Individual concentration-versus-time profiles, linear scale.

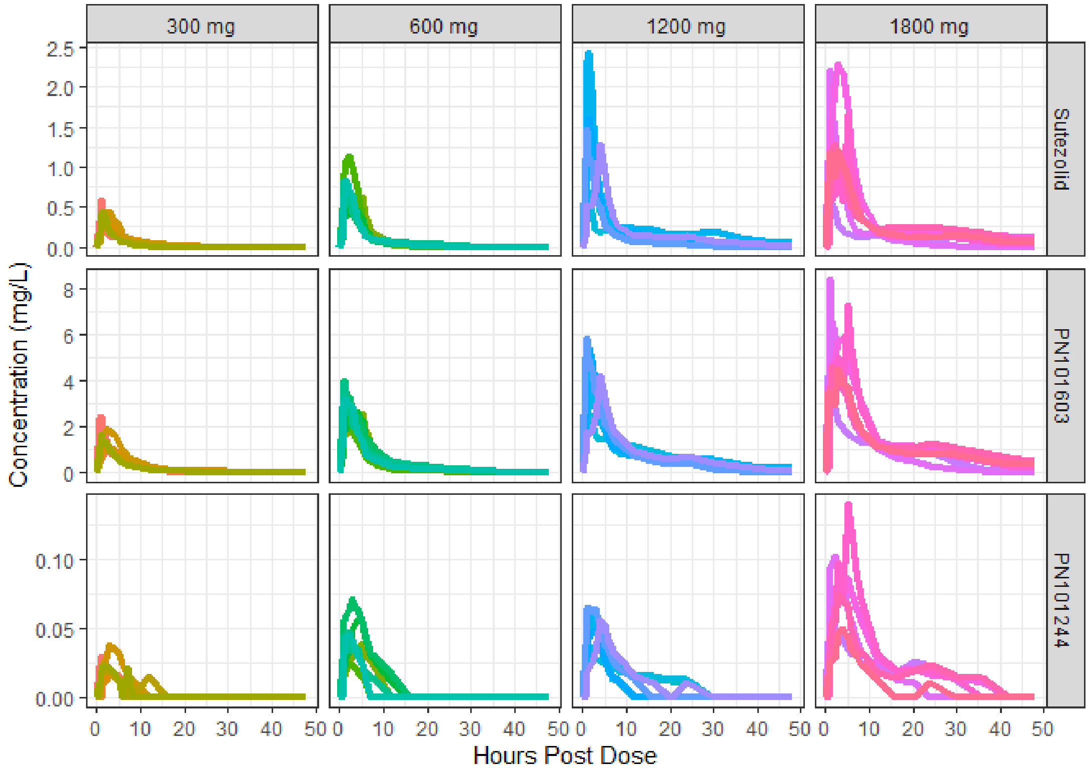

Supplementary Figure S2: Individual concentration-versus-time profiles, log scale.

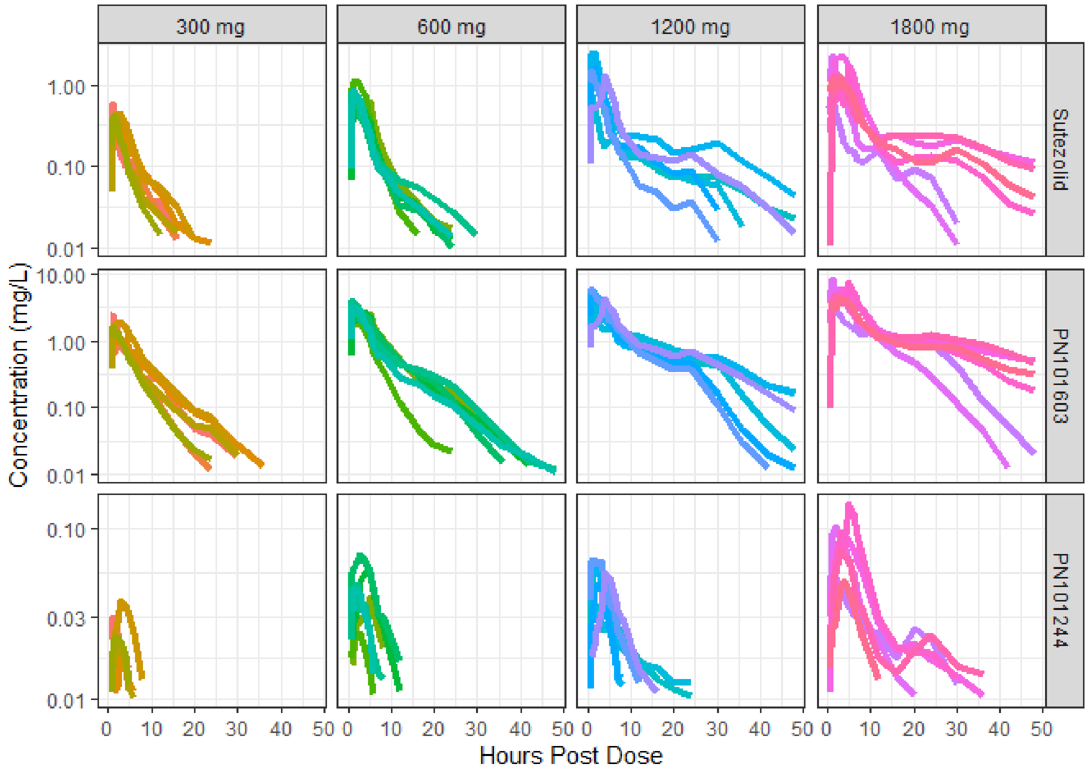

Supplement: Supplemental file 1 — Tables S1 to S3 and Fig. S1 and S2. Download aac.02108-21-s0001.pdf, PDF file, 0.2 MB [file aac.02108-21-s0001.pdf]
